# Comparative study of the effect of two different doses of intravenous labetalol on the cardiovascular response to endotracheal extubation

**DOI:** 10.34172/jcvtr.2023.31623

**Published:** 2023-06-29

**Authors:** Hamidreza Shetabi, Behzad Nazemroaya, Hosein Mahjobipoor, Sanaz Majidi

**Affiliations:** ^1^Anesthesiology and Critical Care Research Center, Isfahan University of Medical Sciences, Isfahan, Iran; ^2^Student Research Committee, Isfahan University of Medical Sciences, Isfahan, Iran

**Keywords:** Extubation, Hemodynamics, Labetalol, Stress Response

## Abstract

**Introduction:**

Providing a stable hemodynamic in extubation is important. We aimed to compare the effect of two different doses of intravenous labetalol on the cardiovascular response to endotracheal extubation.

**Methods:**

This double-blind randomized trial was performed in 2019-2020 in Isfahan on 72 patients under general anesthesia. Patients using Random Allocation software were divided into three groups and received 0.1 mg/ kg or 0.2 mg/kg labetalol and normal saline intravenously 10 min before extubation. Hemodynamic variables including heart rate (HR), Systolic blood pressure (SBP), diastolic blood pressure (DBP), mean arterial pressure (MAP), and peripheral blood oxygen saturation(SPO2) was measured for each patient before induction of anesthesia and 1, 3, 5 and 10 minutes after extubation.

**Results:**

SBP changes were significantly different between the three groups at 1, 3, 5 minutes after extubation (*P*=0.036, *P*=0.009, *P*=0.005 respectively) unlike the other two groups, patients who received 0.2 mg/kg labetalol did not have an increase in DBP after extubation (*P*>0.05). DBP was significantly different between the three groups one minute after extubation (*P*=0.03). At minutes 1 and 3 following extubation, there was a significant difference in the MAP between the three groups. (*P*=0.029 and *P*=0.012 respectively). There was no significant difference between the three groups regarding heart rate (*P*>0.05).

**Conclusion:**

Tracheal extubation is usually associated with an increase in hemodynamic variables. Both doses of labetalol attenuate the hemodynamic response accompanying tracheal extubation. But labetalol 0.2 mg/kg in reducing hemodynamic response to extubation acted more effectively than labetalol 0.1mg/kg.

## Introduction

Tracheal intubation during anesthesia is the standard and reliable way of maintaining airways, and securing pulmonary ventilation.^1^ Intubation and extubation are both associated with different cardiovascular and respiratory responses that lead to tachycardia, hypertension, arrhythmia, myocardial ischemia, cough, bronchospasm, increased bleeding, and increased intracerebral and intraocular pressure.^2^ Hemodynamic responses are caused by sympathoadrenal stimulation due to manipulation of the epipharyngeal and parapharyngeal regions, which leads to a significant increase in catecholamine levels, resulting in increased blood pressure and heart rate.^3,4^

Sympathoadrenal stimulation occurs within 5 seconds after laryngoscopy and increases with the passage of the endotracheal tube reaches a maximum within 1-2 minutes and returns to normal after 5 minutes.^5^ Tracheal extubation is part of the process of general anesthesia with endotracheal intubation.^6^ Complications after extubation are 3 times more common than those that occur during intubation and induction of anesthesia.^7^ Tachycardia and hypertension are common accidents associated with tracheal tube removal.^8^ These hemodynamic responses are triggered by epipharyngeal and laryngeal stimulation (stimulation of the sympathoadrenal reflex) along with a concomitant increase in plasma catecholamine levels and activation of alpha and beta-adrenoceptors.^9^

Extubation leads to a 10% -30% increase in blood pressure and heart rate for 5-15 minutes and in coronary artery disease patients causes a 40% -50% decrease in heart ejection fraction.^10,11^ Careful monitoring and control of blood pressure at the end of surgery and during extubation is very important to prevent hypertension in the recovery room and postoperative bleeding.^12^

A variety of techniques have been used to reduce cardiovascular responses and cough during extubation, including extubation under deep anesthesia and the use of drugs such as narcotics, dexmedetomidine, calcium channel blockers, and lidocaine. ^13^

Labetalol is an adrenergic antagonist antihypertensive agent that acts on selective 1α receptors and non-selective 1β and 2β receptors. It has a fast effect and reaches its peak effect 5 to 15 minutes after intravenous injection. ^14^ Labetalol reduces blood pressure by reducing systemic vascular resistance (1α blockade) without changing cardiac output and reduces reflex tachycardia through β blockade. ^15^ Various studies have investigated the effect of different drugs in reducing the hemodynamic response to intubation and extubation and have reported valuable results.

 Since none of the drugs mentioned yet has a definite effect in reducing the cardiovascular response to extubation, and given the risks that these changes have in patients with coronary artery disease, hypertension, and cerebrovascular disease, finding a drug that is more effective in this area is very important. Also, according to the literature review, no study has been performed to evaluate the effect of two doses of labetalol on extubation. Therefore, in this study, we intend to compare the effect of two different doses of labetalol (0.1 mg/ kg and 0.2 mg/ kg) on ​​the cardiovascular response to extubation.

## Materials and Methods

 This double-blinded randomized clinical trial was performed during the period of 2019-2020 in Al-Zahra Hospital affiliated with Isfahan University of Medical Sciences. The study protocol was approved by the Ethics Committee of Isfahan University of medical sciences with code: IR.MUI.MED.REC.1399.633, registered with ID: IRCT20180416039326N16 in the Iranian Clinical Trials Center (IRCT). The study using easy sampling was conducted on patients that were candidates for elective surgical procedures requiring intubation

 The study was double-blinded, the patient and the observer who collected the data were unaware of the drug grouping.

 Inclusion criteria included age between 20 and 60 years, elective surgery with anticipated intubation time less than 2 hours, American Society of Anesthesiologists (ASA) classification equal to 1 or 2, weight between 55 and 85 kg, and Written informed consent to participate in this study.

 Patients with the following conditions did not enter the study: pregnant women, patients with diabetes, patients with uncontrolled cardiovascular diseases, baseline heart rate lower than 60 per minute, SBP (SBP) lower than 90 mmHg, patients with cerebral vascular diseases, contraindication for the drugs used in this study, allergies to the drugs used in this study, substance abuse and taking medication with cardiovascular effects.

 Exclusion criteria were: occurrence of drug sensitivity, change in anesthetic process, surgery time lasting more than 2 hours, transfer to ICU, and re-intubation during recovery time.

 The sample size was calculated at 26 patients in each group according to the following formula for analysis of variance of repeated measurements with a significant level of 5% (z = 1.96), statistical power 80% (z = 0.84), to detect the standardized effect size of at least Δ = 0.5 for all hemodynamic indices with one observation before (v = 1) and 5 observations after intervention (w = 5) and intra-cluster correlation coefficient of p = 0.6, which leads to R = 0.32.

 Patients who meted the entry criteria were recruited. The blind observer collected demographic data of all cases including age, gender, weight, ASA classification, and presence of any comorbidities.

 Upon entering the operating room, all patients underwent electrocardiogram monitoring, non-invasive intermittent sphygmomanometers, pulse oximetry, and capnography. Hemodynamic variables including heart rate (HR), SBP (SBP), diastolic blood pressure (DBP), mean arterial pressure (MAP), and peripheral blood oxygen saturation were measured for each patient before anesthesia induction as a baseline.

 Induction of anesthesia was performed with propofol (2 mg/kg), fentanyl 2 µg/kg, and then atracurium (0.5 mg/kg). In all patients, after 3 minutes of respiratory support with a mask and ambu bag, intubation was performed. Maintenance of anesthesia was with 50% oxygen and nitrous oxide, isoflurane 1-1.2 mac. and morphine 0.1 mg/kg.

 Patients were then divided into 3 groups using Random Allocation software. The doses used for labetalol have been selected based on previous studies. Previous studies have used higher doses of labetalol in endotracheal intubation. In the present study, lower doses were used in tracheal tube extubation:

 Group 1 received 0.1 mg/ kg labetalol diluted in 0.9% normal saline up to 5 ml.

 Group 2 received 0.2 mg/ kg labetalol diluted in 0.9% normal saline up to 5 ml.

 Grope 3 received 5 ml of 0.9% normal saline.

 At the end of the surgery, the anesthetic gases were cut off, and reversing the relaxants was conducted using atropine 0.02mg/ kg and neostigmine 0.04 mg/ kg, the patients received 3 liters of oxygen per minute, and the extubation was performed.

 The hemodynamic variables were measured before extubation, 1, 3, 5, and 10 minutes after exiting the endotracheal tube. Complications of extubation including adverse airway responses (cough, laryngospasm, bronchospasm, hypoxemia, bradycardia, and apnea) and hemodynamic complications (tachycardia, bradycardia, hypertension, and hypotension) were evaluated, treated, and recorded based on the patient’s clinics. Also, the length of stay in the recovery was recorded using the modified Aldrete score 9.

 The quantitative variable was described by mean and standard deviation (SD) and categorical variables using frequency and percentage. The normality of the continuous variable was assessed by the Kolmogorov-Smirnov test. Continues variables between arms were compared using analysis of variance or the Kruskal-Wallis test. The frequency of categorical data was compared using Chi-square or Fisher’s exact tests. The change of main outcome including BP, MAP, PR, and O2 saturation (O2 Sat) during the procedure was compared using repeated measure analysis of variance. Statistical analyses were conducted by SPSS, version 23. P values less than 0.05 was considered statistically significant.

## Results

**Figure 1:**
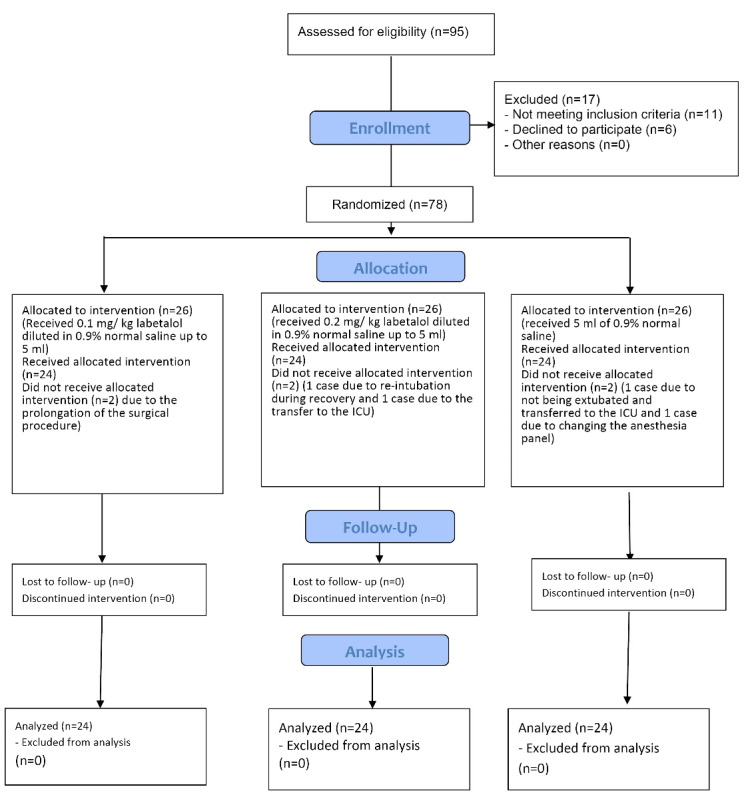
The CONSORT flowchart of patients

In the current study, out of 78 patients studied, 2 patients in the labetalol 0.2 mg/kg group (1 case due to re-intubation during recovery and 1 case due to the transfer to the ICU), 2 patients in the labetalol 0.1mg/kg group (due to the prolongation of the surgical procedure) and 2 patients in the control group (1 case due to not being extubated and transferred to the ICU and 1 case due to changing the anesthesia panel) were excluded from the study. So, the study was analyzed on three groups of 24, flowchart of the patients selection (CONSORT flow diagram) is illustrated in Figure 1.

The primary analysis of demographic data showed that there were no significant differences between the three groups regarding age, weight, gender, and ASA class (*P* > 0.05). These data are summarized in Table 1.

**Table 1:** Basic and clinical characteristics of patients in the three groups

**Variables**		**Labetalol 0.2 mg/kg group(n=24)**	**Labetalol 0.1mg/kg group(n=24)**	**Control group(n=24)**	***P*** ** value**
Age; years		37.2 ± 10.4	31.79 ± 10.55	33 ± 14.75	0.24
Weight; Kg		71.04 ± 7.37	72.92 ± 9.22	69.9 ± 9.84	0.35
Sex	Male	7(29.2%)	13(54.2%)	5(20.8%)	0.73
	Female	17(70.8%)	11(45.8%)	19(79.2%)	
ASA class	1	20(83.3)	10(28.6%)	17(77.3)	0.87
	2	4(16.7)	25(71.4%)	5(22.7)	

Data shown Mean ± SD or n(%). ASA class: American Society of Anesthesiologists (ASA) classification

Evaluation of hemodynamic parameters showed that the SBP was significantly different between the three groups at 1, 3, and 5 minutes after extubation (*P* = 0.036, *P* = 0.009, P = 0.005 respectively) and patients that received labetalol 0.2mg/kg had the least changes compared to other groups (*P* > 0.05). Unlike the other two groups, patients who received 0.2 mg/kg labetalol did not have an increase in DBP after endotracheal tube removal (*P* > 0.05).

Comparison of groups showed that the mean changes in SBP(SBP) between the two groups of labetalol 0.2 mg/kg, 0.1 mg/kg (*P* = 0.007) and the control group (*P* = 0.035) compared to baseline SBP was significant. There was no significant difference between the two groups of 0.1 mg/kg and the control group, despite lower blood pressure and heart rate (*P* = 0.90).

Evaluations mean of diastolic blood pressure(DBP) showed that group 0.2mg/kg labetalol was associated with an attenuated response to extubation with a minimal diastolic blood pressure change, but there was a significant difference between the three groups in 1 minute after extubation(*P* = 0.03).

The evaluation of the arterial pressure during the study period showed that labetalol 0.2mg/kg was more effective than labetalol 0.1mg/kg in attenuating the response to endotracheal tube extubation and MAP changes were less, but at 1 and 3 minutes after extubation MAP changes were significant between the three groups. (*P* = 0.029 and *P* = 0.012 respectively).

There was no significant difference between the three groups in monitoring heart rate, despite the increase in heart rate to extubation in group 0.1mg/kg and placebo compared to 0.2mg/kg (*P* = 0.053). In comparison between the two groups, heart rate changes between the two groups of labetalol were significant (*P* = 0.02), but the difference between the groups of labetalol 0.2 mg/kg and labetalol 0.1 mg/kg (*P* = 0.14) and labetalol 0.1 mg/kg and control group (*P* = 0.97) were not significant. The percentage of blood oxygen saturation was not significantly different between the three groups, but in the intragroup study, the trend of SPO2 changes was significant only in the labetalol 0.1 mg/kg group. In comparison between groups, the trend of SPO2 changes was not significantly different between the three groups (*P* = 0.20). These data are indicated in Table 2.

**Table 2:** Comparison of hemodynamic parameters of patients in the three groups

**Variables**	**Time**	**Labetalol 0.2 mg/kg group(n=24)**	**Labetalol 0.1mg/kg group(n=24)**	**Control group(n=24)**	***P*** ** value**
SBP (mmHg)	Before anesthesia	120.4 ± 7.2	122.1 ± 11.6	122 ± 8.8	0.10
	Before extubation	125.7 ± 12.2	131.2 ± 15.9	123.8 ± 16.4	0.22
	1 minute	126.7 ± 14.6	19 ± 137.1	20 ± 139.3	0.036
	3 minutes	122.7 ± 13	18.1 ± 136	16 ± 134.3	0.009
	5 minutes	9.4 ± 117.9	21.2 ± 131.4	15.2 ± 132.7	0.005
	10 minutes	15 ± 125	16.7 ± 132.4	17 ± 132.1	0.21
	P**	0.001 >	0.001 >	0.001 >	0.03^***^
Diastolic blood pressure (mmHg)	Before anesthesia	4.5 ± 74.3	7.9 ± 75.5	9.6 ± 77.00	0.72
	Before extubation	8.7 ± 77.9	14.8 ± 81.9	12.1 ± 77.6	0.38
	1 minute	12.3 ± 78.4	12.9 ± 86.1	12.3 ± 84.4	0.03
	3 minutes	12.2 ± 77.5	12.3 ± 82.5	12.5 ± 81.9	0.23
	5 minutes	13 ± 75.2	10.8 ± 78.9	11 ± 80.3	0.31
	10 minutes	11 ± 77.3	12.3 ± 79.8	12.3 ± 78.6	0.85
	P**	0.23	0.001	0.004	0.64^***^
Mean arterial pressure (mmHg)	Before anesthesia	5.03 ± 105.7	2.9 ± 106.5	8.6 ± 106.2	0.4
	Before extubation	9.1 ± 109.7	16.9 ± 111.1	14.1 ± 108.4	0.31
	1 minute	11.9 ± 10.6	16.5 ± 120.1	16.3 ± 121.2	0.029
	3 minutes	11.1 ± 107.7	15.1 ± 118.2	13.5 ± 117.1	0.012
	5 minutes	12.8 ± 108.5	14.5 ± 115.8	14 ± 114.8	0.13
	10 minutes	14 ± 96.2	15.8 ± 95.7	13.2 ± 97.4	0.93
	P**	0.001 >	0.001 >	0.001 >	***0.041
Heart rate (bpm)	Before anesthesia	7.7 ± 80.15	10.7 ± 83.2	6.4 ± 85.6	0.11
	Before extubation	10.2 ± 83.1	12.4 ± 86.2	13.6 ± 85/0	0.64
	1 minute	11.7 ± 84.7	14.5 ± 93.6	15.1 ± 91.3	0.08
	3 minutes	11.4 ± 81.5	13.1 ± 82.6	14.7 ± 88.7	0.15
	5 minutes	11.6 ± 79.2	10.5 ± 83.3	12.5 ± 86.6	0.07
	10 minutes	12.6 ± 70.2	10.5 ± 82	12.5 ± 84.2	0.32
	P**	0.036	0.001 >	0.28	***0.053
O2 saturation (%)	Before anesthesia	1.4 ± 98.5	1.2 ± 98.1	1.7 ± 98.1	0.56
	Before extubation	1.1 ± 99.3	0.9 ± 98.3	2.8 ± 98.1	0.07
	1 minute	1.9 ± 98.6	3.2 ± 97.1	2.1 ± 98.2	0.10
	3 minutes	2.2 ± 98.3	1.8 ± 98.1	2.5 ± 97.9	0.80
	5 minutes	1.8 ± 98.6	2.7 ± 98.3	3.8 ± 97.1	0.19
	10 minutes	1.9 ± 98.7	2 ± 98.3	2.7 ± 97.6	0.29
	P**	0.30	0.005	0.36	***0.20

* Significant level of difference between the three groups in each time period according to one-way analysis of variance test ** Significance level of changes within each group during the study period in terms of analysis of variance with repeated observations *** Significance level of changes between the three groups during the study period in terms of analysis of variance with repeated observations

The mean length of stay in recovery in the three groups of labetalol was 0.1 mg/kg, 0.2 mg/kg and the control group was 70.83 ± 14.9, 79.8 ± 24.4, and 80.45 ± 22.9 minutes respectively. In addition, no significant difference was observed between the three groups (*P* = 0.22).

**Figure 2:**
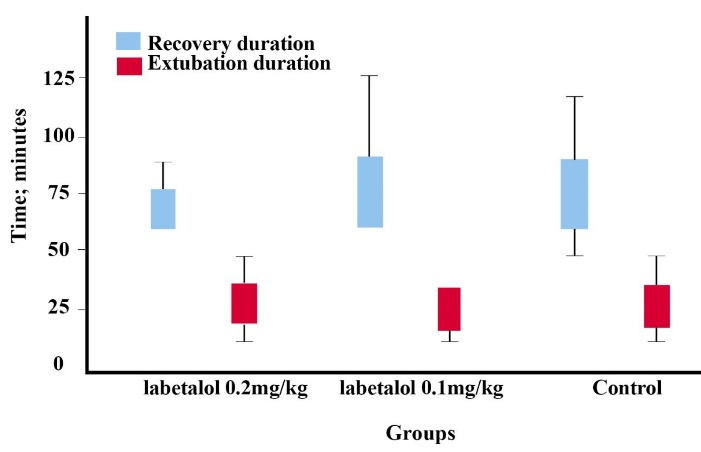
Boxplat for recovery and extubation duration in the three groups

The mean extubation time in the three groups was 21.46 ± 12.81, 19.8 ± 15.83, and 22.86 ± 15.34 minutes respectively, and there was no significant difference between the three groups (*P* = 0.59) (Figure 2 ).

 The extubation time was considered from the time of stopping the anesthetic agent to the immediate removal of the endotracheal tube.

During the intervention, 23 patients developed airway disorders, including 6 in the labetalol 0.2 mg/kg group, 8 in the 0.1 mg/kg group, and 9 in the control group (25%, 33.3%, and 40.9%, respectively). There was no significant difference between the three groups (*P* = 0.52). The type of complications included 22 cases of cough and 1 case of hypoxia. The incidence of cough in the three groups of 0.2 mg/kg, 0.1 mg/kg, and control were 6, 8, and 8 cases (25%, 33.3%, and 36.4%, respectively) and no significant difference was observed between the three groups (*P* = 0.73). The only case of hypoxia was in the control group (4.5%) (*P* = 0.31).

The incidence of hemodynamic disorders in the three groups of labetalol 0.2 mg/kg, labetalol 0.1 mg/kg, and control were 4, 11, and 10 cases (16.7%, 45.8%, and 45.5%, respectively). However, the difference between the three groups was not significant (P = 0.051). The types of hemodynamic disorders included tachycardia, hypotension, and hypertension, the frequency of which is shown in Table 3.

**Table 3:** Complications and adverse cardiovascular response in three groups

**Complications**		**Labetalol 0.2 mg/kg group(n=24)**	**Labetalol 0.1mg/kg group(n=24)**	**Control group(n=24)**	***P*** ** value**
Respiratory tract complication	Cough	6(25%)	8(33.3%)	8(36.4%)	0.73
	Hypoxia	0(0%)	0(0%)	1(4.5%)	0.31
Hemodynamic disorders	Tachycardia	1(4.2%)	6(25%)	5(22.7%)	0.098
	Hypotension	1(4.2%)	0(0%)	0(0%)	-
	Hypertension	2(8.3%)	5(20.8%)	5(22.7%)	0.891

According to Chi-square test, the type of hemodynamic disorder was not significantly different between the three groups (*P* = 0.098). None of the patients required re-intubation during the study period.

## Discussion

 In this study, we compared the hemodynamic changes in patients undergoing tracheal tube extubation in three groups receiving labetalol 0.2mg/kg, labetalol 0.1mg/kg, and saline, the result showed that at 1, 3, and 5 minutes after extubation, SBP changes were significantly different between the three groups, in patients that received labetalol 0.2mg/kg had the least changes compared to other groups and in labetalol 0.2mg/kg group unlike the other two groups, patients did not have an increase in DBP after endotracheal tube removal. Evaluations mean of diastolic blood pressure showed that group 0.2mg/kg labetalol was associated with a more attenuated response to extubation with a minimal diastolic blood pressure change, but there was a significant difference between the three groups in 1 minute after extubation. Evaluation of MAP during the study period showed that labetalol 0.2mg/kg was more effective than labetalol 0.1 mg/kg in attenuating the response to endotracheal tube extubation and MAP changes were less, At 1 and 3 minutes after extubation, MAP changes in the other two groups compared to the 0.2mg/kg group were statistically significant

 Despite the increase in heart rate to extubation in the 0.1mg/kg group and placebo compared to 0.2mg/kg, there was no significant difference between the three groups. Our study shows that both doses of labetalol attenuate hemodynamic responses to extubation. The attenuation of hemodynamics responses to tracheal extubation in the labetalol group 0.2 was greater than 0.1.

 There have been previous studies that evaluated the effects of labetalol on hemodynamics in different situations.

One of the findings of our study was significantly lower and more stabilized SBP in patients receiving higher dosages of labetalol. Patel and colleagues conducted a study on 60 patients undergoing extubation and compared the hemodynamic changes of patients receiving esmolol 1.5 mg/kg and labetalol 0.1 mg/kg. The results indicated that both esmolol and labetalol attenuated hemodynamic response and patients with labetalol had a significant decrease in the SBP.^16^

Younes et al conducted a study on 80 patients in 4 groups of 20. 15 minutes before tracheal tube removal, patients received either labetalol 0.25 mg/kg, lidocaine 2% 1.5 mg/kg, fentanyl 2µg/kg, or normal saline. They stated in the results, both labetalol and fentanyl effectively blunt hemodynamic response to tracheal extubation and can be safely used. Labetalol at a dose of 0.25 mg/kg is more effective than fentanyl and lidocaine in reducing the hemodynamic response to tracheal extubation.^17^ The results of our study were in line with the findings that Indicate the effectiveness of labetalol in reducing SBP changes to extubation. Kewalramani and colleagues performed a study on 90 patients in three groups, who received 0.5mcg/kg dexmedetomidine, 0.25mg/kg labetalol, or 0.9% normal saline. in conclusion, hemodynamic responses to laryngoscopy, endotracheal intubation, and extubation were better controlled with dexmedetomidine than labetalol.^18^ An important point is that we compared the effectiveness of two different dosages of labetalol (0.1mg/kg and 0.2mg/kg) and our data showed higher effectiveness for 0.2mg/kg dosage while in the previous studies, only one dosage had been assessed. Another point is that these data highlight the use of labetalol in patients undergoing extubation which indeed provides more stabilized hemodynamics and lower SBP compared to a placebo.

Another finding of this study was to reduce heart rate changes following extubation in patients receiving labetalol, especially at a dose of 0.2mg/kg. El-Shmaa and others showed that administration of dexmedetomidine was associated with more stable hemodynamics compared to labetalol but they also declared that labetalol resulted in significantly reduced PR.^19^ Similar results were also indicated by Xue-jun and colleagues. In their study, it was conferred that decreased PR was associated with a decrease in SBP that provided significantly stable hemodynamics in patients during tracheal extubation.^20^ These data were also in line with the findings of our study but we should note that these studies have used 0.1mg/kg of labetalol but no comparisons have been made with other dosages.

Singla and colleagues assessed 160 borderline hypertensive patients that underwent laparoscopic cholecystectomy. It was reported that administration of labetalol 0.1mg/kg did not make differences in hemodynamics including SBP and PR while the use of dexmedetomidine had more significant results in this regard.^21^ These differences are due to the variations in the characteristics of the study population as the mentioned study was specifically conducted on patients with borderline hypertension.

Furthermore, one of the other findings of our study was lower rates of hemodynamic complications in the group of patients that received labetalol 0.2mg/kg. This finding also emphasizes the efficacy of 0.2mg/kg labetalol in patients undergoing extubation. Based on the findings of Attari and colleagues, labetalol had fewer hemodynamic complications compared to morphine during emergence from anesthesia after craniotomy.^22^

Ratnani and his colleagues in a trial concluded that labetalol 0.25mg/kg is an effective and safe drug to reduce sympathomimetic responses to tracheal intubation, while esmol 0.5mg/kg and lignocaine 1mg/kg were somewhat effective and safe.^23^

 The results of the above studies were in line with the findings of our study. The limitations of the present study were the restricted study population and that we did not include patients that underwent a single type of surgery. These items might be important and effective on types of hemodynamic responses in patients. Altogether, we conclude that 0.2mg/kg labetalol was more effective than 0.1mg/kg labetalol in providing stabilized hemodynamics during extubation. We recommend that anesthesiologists should pay more attention to the properties of 0.2mg/kg labetalol.

## Conclusion

 Tracheal extubation is usually associated with an increase in hemodynamic variables. Both doses of labetalol attenuate the hemodynamic response accompanying tracheal extubation. But labetalol 0.2mg/kg in reducing hemodynamic response to extubation acted more effective than labetalol 0.1 mg/kg.

## Acknowledgements

 The authors appreciate the good cooperation of the anesthesia personnel and the nurses of the recovery department of the operating room of Al-Zahra Hospital.

## Competing Interests

 The authors declare no conflicts of interest.

## Ethical Approval

 The protocol of this study was approved by the ethics committee of the Isfahan University of Medical Sciences with the ethics code IR.MUI.MED.REC.1399.633.

## Funding

 This study was supported by the Isfahan University of Medical Sciences fund with code 8512.
